# Development and Performance Evaluation of a Core–Shell Structure Gel Plugging Agent for Ultra-High-Temperature and High-Salinity Water-Based Drilling Fluids

**DOI:** 10.3390/gels12050446

**Published:** 2026-05-19

**Authors:** Yuhao Xia, Fengfeng Xiao, Jun Wang, Jingping Liu, Meng Li, Yuanwei Sun

**Affiliations:** 1 CNPC Chuanqing Drilling Engineering Co., Ltd., Chengdu 610051, China; xiayuhao_sc@cnpc.com.cn (Y.X.); xiaoff_sc@cnpc.com.cn (F.X.); wangjun1_sc@cnpc.com.cn (J.W.); 2 School of Petroleum Engineering, China University of Petroleum—East China (UPC), Qingdao 266580, China; liujingping20@126.com (J.L.); z25020060@s.upc.edu.cn (M.L.)

**Keywords:** gel plugging agent, water-based drilling fluid, high-temperature and salt resistance, core–shell structure

## Abstract

Gel plugging agents are key drilling fluid additives for maintaining wellbore stability. However, the downhole ultra-high-temperature, high-salinity environments, and developed micro-fractures in deep and ultra-deep wells pose severe challenges to the performance of gel plugging agents. To address this problem, this paper presents the preparation of a nano-micron gel plugging agent with a core–shell structure, denoted as LMS, suitable for high-temperature and high-salinity water-based drilling fluids. LMS was synthesized via emulsion polymerization, using a styrene–sodium p-styrenesulfonate copolymer as the core and 2-acrylamido-2-methylpropanesulfonic acid and methacryloyloxyethyltrimethyl ammonium chloride as the shell-modifying monomers. LMS was characterized by infrared spectroscopy, thermogravimetric analysis, transmission electron microscopy, and particle size analysis, confirming that LMS met the design expectations. Experimental results showed that after aging at 220 °C for 16 h under saturated-salt conditions, the filtration loss of the drilling fluid with 3 wt% LMS was 10.4 mL, a reduction of 57.4% compared to the base mud. Meanwhile, LMS exhibited good plugging performance in microporous membrane tests and sand bed tests. After aging at 220 °C for 16 h under saturated-salt conditions, the core plugging rate reached 95.4%. LMS can not only adsorb onto clay surfaces to increase the thickness of the hydration film, enhancing drilling fluid stability, but can also synergistically build a filter cake with clay particles to plug nano-micron pores, preventing drilling fluid infiltration into the formation. This paper provides a preparation method for a high-temperature- and high-salinity-resistant gel plugging agent with excellent plugging effects, which is expected to support safe and efficient drilling in deep and ultra-deep formations.

## 1. Introduction

As oil and gas development advances towards deep and ultra-deep formations [1], the ultra-high temperature and high-pressure environment in deep strata imposes higher requirements on drilling fluid performance [2,3]. When drilling through fractured or micro-fractured formations, severe lost circulation of drilling fluid into the formation can easily occur, potentially leading to wellbore instability or even collapse [4]. Drilling fluid gel plugging agents are crucial additives to address these issues [5,6]. The plugging methods of plugging agents mainly include physical methods and chemical methods. The physical method mainly uses inert materials such as particles and fibers to directly plug the loss channels, such as bridge plugging with added walnut shells and calcium carbonate. The chemical method mainly involves injecting materials, such as cement or gel, which undergo chemical reactions in the formation and then solidify, forming a high-strength seal, such as cement consolidation and polymer crosslinking [7,8]. Water-based drilling fluids are among the most commonly used types in oilfields due to their environmental friendliness, safety, cost-effectiveness, and highly tunable performance [9,10]. Therefore, developing a water-based drilling fluid gel plugging agent resistant to ultra-high temperatures and high salinity is crucial for deep and ultra-deep well drilling.

In recent years, significant progress has been made in the research of water-based drilling fluid gel plugging agents [11,12]. Currently, water-based drilling fluid gel plugging agents are mainly divided into asphalt-based and nanomaterial-based types [13]. Asphalt is widely used as a plugging material in drilling fluids to maintain wellbore stability, primarily in hard brittle shale formations with developed micro-fissures [14]. Yang et al. [15] studied a new modified emulsified asphalt for plugging micro-fractures, achieving a softening point of 180 °C at 5% dosage, enabling adaptive plugging of nano- and micron-scale fractures. However, the temperature and salt resistance of asphalt-based gel plugging agents are gradually failing to meet the demands of increasing drilling depths, and their poor environmental friendliness limits their use in areas with high environmental requirements [16,17]. Nanomaterials have become a highly promoted type of gel plugging agent in recent years [18,19]. Current water-based drilling fluid nano-plugging materials are divided into inorganic nano-plugging materials and organic nano-plugging materials [20,21]. The mechanism of inorganic nano-plugging materials involves forming physical barrier layers through accumulation and bridging within fractures [22,23]. Song et al. [24] showed that nano-SiO_2_ can effectively seal the formation of micro-fractures by relying on its rigidity, with temperature resistance up to 120 °C and salt resistance up to saturation; 1.5% nano-SiO_2_ particles reduced the filtration loss of the base mud from 34.5 mL to 23 mL. However, inorganic materials do not have deformability, and their size is difficult to change. However, the sizes of formation fractures are often diverse, and in field applications, size compounding is often required. Additionally, they are prone to agglomeration under high-temperature conditions, preventing effective entry into fractures and thus losing their plugging effect [25,26]. The mechanism of organic nanomaterials involves forming dense adaptive adsorption fillings in fractures through their strong adsorption and deformable characteristics, achieving plugging and wellbore stabilization [27]. Tchameni et al. [28] synthesized a novel thermoresponsive Janus nanosilica (TRJS) with unique two-dimensional ion characteristics, achieving temperature resistance up to 200 °C and salt resistance up to saturation. Dong et al. [29] synthesized a terpolymer microsphere (AAK), which could effectively seal nano-pores in rock blocks, reducing the filtration loss of the base mud to 17 mL at a 1.5% dosage, with NaCl resistance up to 7.5%. However, its temperature resistance was only up to 100 °C; insufficient temperature resistance is a common drawback of most current organic nanomaterial gel plugging agents. Furthermore, the plugging layers formed by organic nanomaterials often have weak pressure resistance and can be easily pushed deep into the formation under differential pressure, failing to form an effective seal. In summary, existing water-based drilling fluid plugging agents can achieve maximum temperature resistance up to 200 °C and salt resistance up to 15%, but each type has its own advantages and disadvantages. Under the more demanding conditions of ultra-high temperature (exceeding 200 °C) and high salinity in ultra-deep formations, achieving stable and effective plugging remains challenging [6].

Addressing the above issues, a core–shell-structured nano-micron gel plugging agent, LMS, was designed and developed for high-temperature and high-salinity water-based drilling fluids. LMS possesses the advantages of both flexible and rigid plugging agents. Specifically, its internal core structure exhibits rigidity, which provides effective support, while the external shell structure features good flexibility, offering strong adaptability to micro-pores of varying sizes. A series of characterizations including FTIR, TGA, particle size distribution, and microscopic morphology were conducted on LMS. The effects of LMS on the rheological and filtration properties, as well as plugging performance, of the base mud under high-temperature and high-salinity conditions were studied. The mechanism of LMS was revealed through zeta potential analysis, particle size analysis, mud cake permeability analysis, and scanning electron microscopy.

## 2. Results and Discussion

### 2.1. Characterization

#### 2.1.1. FTIR Analysis

**Figure 1:**
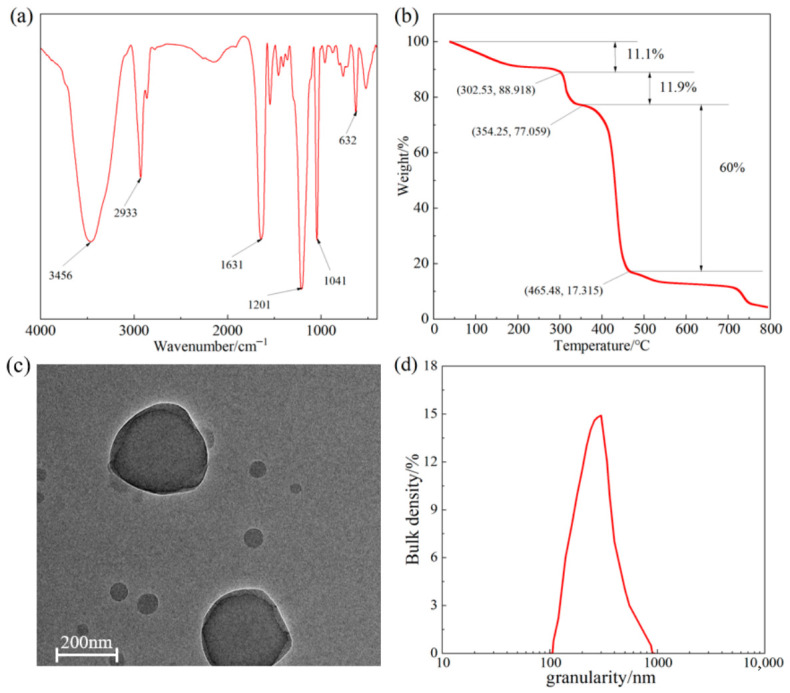
Characterization of LMS: (**a**) FTIR spectrum, (**b**) TGA curve, (**c**) TEM image, and (**d**) particle size distribution.

The FTIR spectrum of LMS is shown in Figure 1a. The absorption peak at 632 cm^−1^ corresponds to the C-S bending vibration [30]. The peak at 1041 cm^−1^ is attributed to the SO^3−^ vibration [31]. The peak at 1201 cm^−1^ corresponds to the C-O bending vibration [32]. The resonance absorption peak at 1631 cm^−1^ is assigned to C=O stretching [33]. The peak at 2933 cm^−1^ is due to the -CH_2_- stretching vibration [34], and the peak at 3456 cm^−1^ corresponds to the N-H stretching vibration [35]. These results confirm that LMS is the target product.

#### 2.1.2. TGA

The TGA curve of LMS is shown in Figure 1b. The weight loss process of LMS is clearly divided into three stages. The first stage ranges from 40 °C to 302.5 °C, where the curve is relatively flat, with a mass loss of approximately 11.1%. This stage is mainly attributed to the volatilization of free water adsorbed by hydrophilic groups in LMS and partial decomposition of side groups in the shell structure, with minimal change in the main LMS structure. The second stage ranges from 302.5 °C to 465.48 °C. As the temperature continues to rise, functional groups in the molecular chains begin to thermally decompose, and both the main and side chains break. In this stage, LMS mass loss is approximately 71.9%, indicating that the main structure of LMS has been destroyed. The third stage occurs after 465.48 °C, where the thermal weight loss gradually slows down, indicating that LMS has been decomposed, leaving some carbonized products. Overall, the thermal decomposition of LMS starts at 302.5 °C, demonstrating good thermal stability.

#### 2.1.3. TEM Analysis

The TEM image of LMS is shown in Figure 1c. LMS exhibits an obvious core–shell structure, consistent with the molecular structure design, with a particle size of approximately 300 nm.

#### 2.1.4. Particle Size Analysis

The particle size distribution curve of LMS at room temperature is shown in Figure 1d. The particle size distribution of LMS is relatively concentrated, ranging from 200 to 500 nm, with a median particle size of 311 nm, corresponding well with the TEM morphology.

### 2.2. Evaluation of Rheological and Filtration Properties

#### 2.2.1. Effect of Gel Plugging Agents on Rheological and Filtration Properties of Base Mud

**Figure 2:**
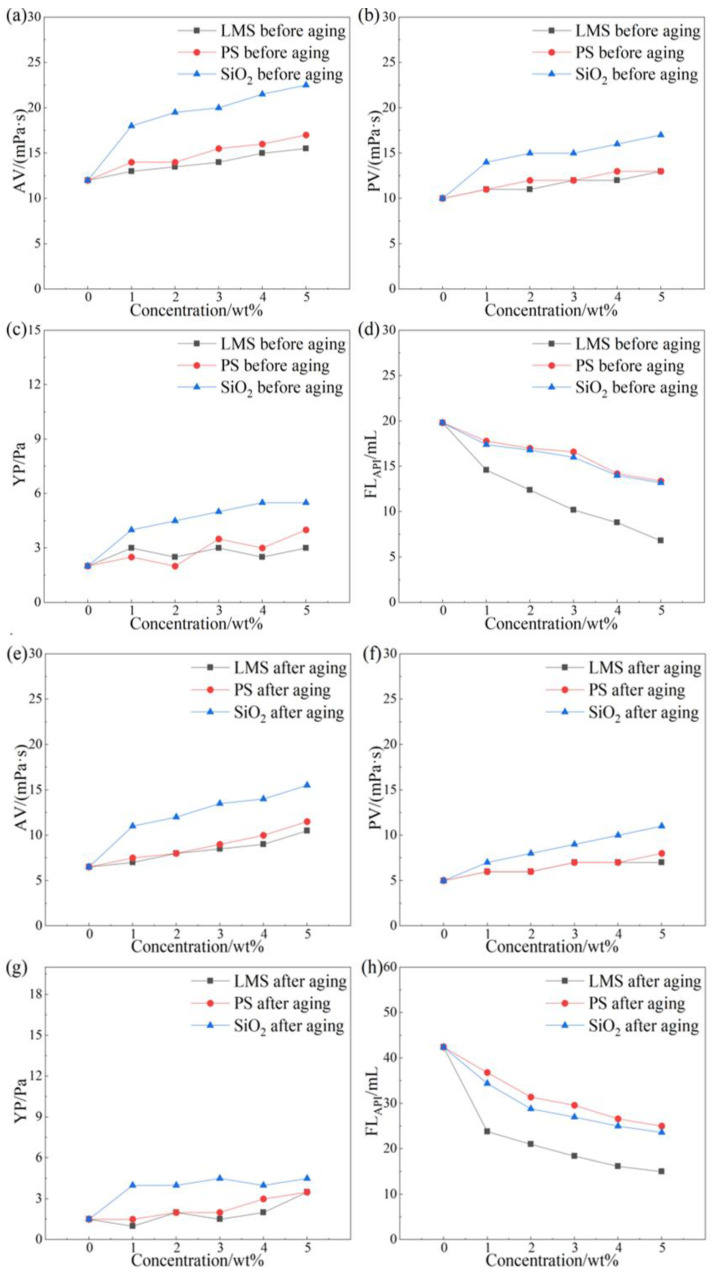
Effect of gel plugging agents on rheological and filtration properties of drilling fluid before aging: AV (**a**), PV (**b**), YP (**c**), and FL_API_ (**d**); after aging at 220 °C for 16 h: AV (**e**), PV (**f**), YP (**g**), and FL_API_ (**h**).

The rheological and filtration properties of the drilling fluid base mud before and after aging with different dosages of LMS, PS, and SiO_2_ are shown in Figure 2.

All three gel plugging agents had some effect on the rheological properties of the base mud before and after aging. SiO_2_ exhibited the strongest viscosity-increasing effect on the base mud, while LMS showed the weakest viscosity-increasing effect. At room temperature, the filtration loss of the base mud was 19.8 mL. All three gel plugging agents reduced the filtration loss of the base mud. When the dosage of all three gel plugging agents was 3 wt%, LMS showed the best filtration reduction effect compared to the base mud, reducing the filtration loss to 10.2 mL, a relative reduction of 48.5%. SiO_2_ showed the next best effect, reducing filtration loss to 16 mL (19.2% reduction). PS showed the poorest filtration reduction effect, with filtration loss reduced to 16.6 mL (16.8% reduction).

After aging at 220 °C for 16 h, the filtration reduction trend of the three gel plugging agents on the base mud remained consistent with that before aging; all exhibited filtration reduction effects. The filtration loss of the aged base mud was 42.4 mL. When the dosage of all three gel plugging agents was 3 wt%, LMS still showed the best filtration reduction effect compared to the base mud, reducing the filtration loss to 18.4 mL, a relative reduction of 56.6%. SiO_2_ showed the next best effect, reducing filtration loss to 27 mL (36.3% reduction). PS showed the poorest filtration reduction effect, with filtration loss reduced to 29.6 mL (30.2% reduction).

Thus, compared to SiO_2_ and PS, LMS has the least impact on the rheological properties of the base mud and exhibits the best filtration reduction effect. Even after aging at 220 °C for 16 h, LMS maintains a good filtration reduction effect compared to SiO_2_ and PS. When the dosage of LMS is 4 wt% and 5 wt%, the gel plugging agent has a stronger thickening effect on the base slurry and has a greater impact on the rheology of the drilling fluid. Additionally, taking into account industrial economic benefits, 3 wt% is the optimal dosage of LMS.

#### 2.2.2. Evaluation of High-Temperature Resistance of Gel Plugging Agents

**Figure 3:**
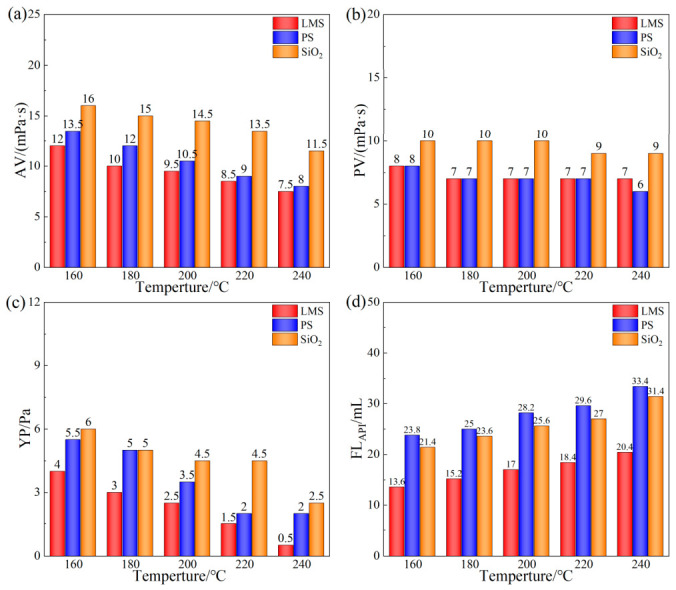
Evaluation of temperature resistance of gel plugging agents. After aging at different temperatures for 16 h: AV (**a**), PV (**b**), YP (**c**), and FL_API_ (**d**).

The rheological and filtration properties of the base mud with 3 wt% LMS, PS, and SiO_2_ after aging at different temperatures for 16 h are shown in Figure 3.

As the aging temperature increased, the viscosity of the base mud containing all three gel plugging agents showed a decreasing trend, while the API filtration loss showed an increasing trend. Compared to SiO_2_ and PS, the base mud containing LMS exhibited the smallest change in rheological properties. When the aging temperature was 240 °C, compared to room-temperature conditions, the filtration loss of the base mud containing PS changed the most, increasing from 16.6 mL to 36.2 mL. The base mud containing SiO_2_ showed an intermediate change, with filtration loss increasing from 16 mL to 33.8 mL. The base mud containing LMS showed the smallest change in filtration loss, increasing from 10.2 mL to 19.2 mL.

Thus, compared to SiO_2_ and PS, LMS performs better under high-temperature conditions, maintaining good performance even at 240 °C.

#### 2.2.3. Evaluation of Salt Resistance of Gel Plugging Agents

**Figure 4:**
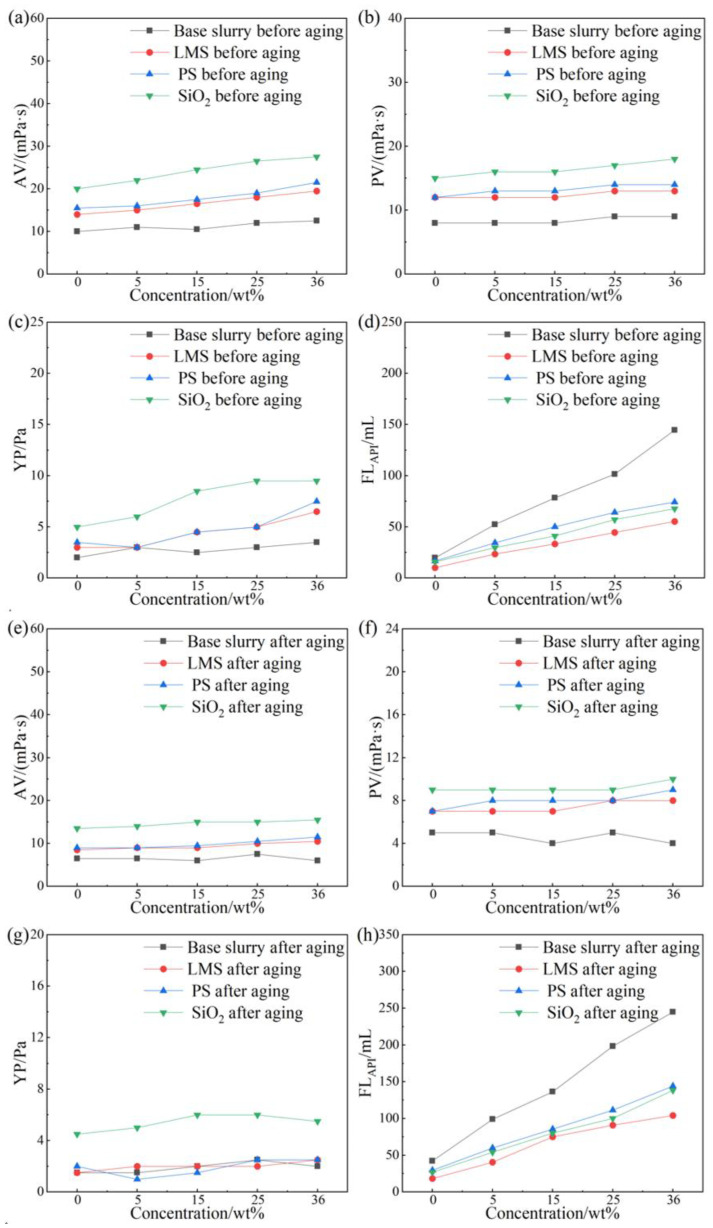
Evaluation of salt resistance of gel plugging agents. Before aging: AV (**a**), PV (**b**), YP (**c**), and FL_API_ (**d**); after aging at 220 °C for 16 h: AV (**e**), PV (**f**), YP (**g**), and FL_API_ (**h**).

The rheological and filtration properties of the drilling fluid with 3 wt% LMS, PS, and SiO_2_ at different NaCl concentrations before and after aging are shown in Figure 4.

With increasing NaCl concentration, the viscosity of the base mud containing 3 wt% of the three gel plugging agents increased to a certain extent both before and after aging, but the overall change was relatively small. At room temperature, as the NaCl concentration increased, the filtration loss of the base mud containing 3 wt% of the three gel plugging agents increased. At a NaCl concentration of 36 wt%, the filtration loss of the base mud was 144.6 mL. Compared to the base mud, the filtration loss of the base mud with LMS decreased the most, to 55.4 mL, a relative reduction of 61.7%. The base mud with SiO_2_ showed an intermediate reduction, to 67.8 mL (53.1% reduction). The base mud with PS showed the smallest reduction, to 74.3 mL (48.6% reduction).

After aging at 220 °C for 16 h, with increasing NaCl concentration, the filtration loss trend for the base mud with 3 wt% of the three gel plugging agents remained consistent with that before aging, with all samples showing increases. At a NaCl concentration of 36 wt%, the filtration loss of the base mud was 244.8 mL. Compared to the base mud, the filtration loss of the base mud with LMS decreased the most, to 104.2 mL, a relative reduction of 57.4%. The base mud with SiO_2_ showed an intermediate reduction, to 138.4 mL (43.5% reduction). The base mud with PS showed the smallest reduction, to 144.2 mL (41.1% reduction).

Thus, compared to SiO_2_ and PS, LMS exhibits superior filtration reduction performance under high-temperature and high-salinity conditions.

### 2.3. Evaluation of Plugging Performance

#### 2.3.1. Microporous Membrane Plugging Evaluation

**Figure 5:**
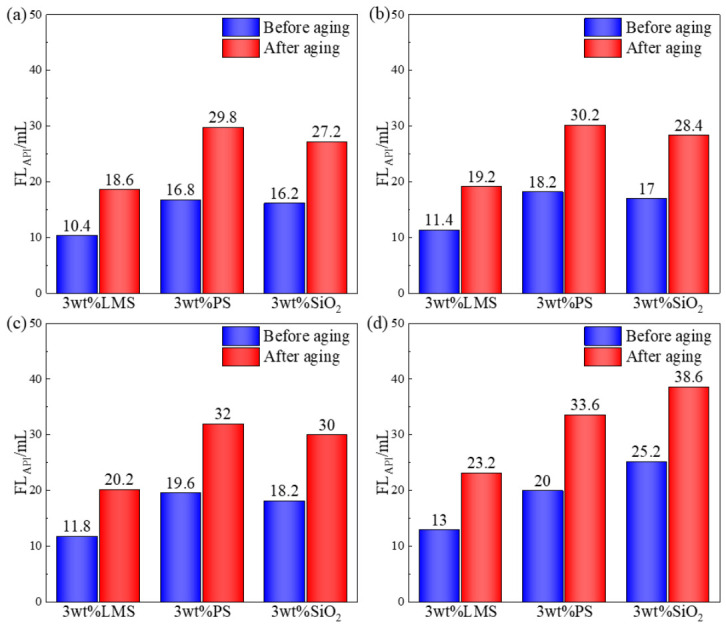
Evaluation of microporous membrane plugging performance: (**a**) PTFE-100, (**b**) PTFE-300, (**c**) PTFE-500, and (**d**) PTFE-1000.

The API filtration loss results of 3 wt% LMS, PS, and SiO_2_ aqueous solutions on PTFE microporous membranes with different pore sizes, before and after aging at 220 °C, are shown in Figure 5.

The experimental results show that LMS, PS, and SiO_2_ can all fill the pores of the microporous membrane to some extent, thereby reducing the room temperature API filtration loss. Specifically, for the PTFE microporous membrane with a pore size of 100 nm, at room temperature, PS exhibited the poorest plugging performance with an API filtration loss of 16.8 mL, SiO_2_ showed intermediate performance with 16.2 mL, while LMS demonstrated the best plugging performance, with an API filtration loss of only 10.4 mL. After aging at 220 °C, due to the high-temperature effect, the API filtration losses for LMS, PS, and SiO_2_ increased to 18.6 mL, 29.8 mL, and 27.2 mL, respectively. However, compared to SiO_2_ and PS, LMS still maintained good plugging performance. For PTFE microporous membranes with pore sizes of 300 and 500 nm, the overall trend in filtration loss was consistent with that for PTFE-100. However, for the PTFE microporous membrane with a pore size of 1000 nm, LMS showed the best performance both before and after aging, PS was intermediate, and SiO_2_ performed the worst. This is because the particle size of rigid gel plugging agents needs to match the pore/fracture size. When the pore size is 1000 nm, SiO_2_ cannot achieve adaptive plugging, resulting in the poorest performance. Thus, compared to SiO_2_ and PS, LMS maintains good plugging and filtration reduction performance under high-temperature conditions, indicating its excellent temperature resistance.

#### 2.3.2. Sand Bed Plugging Evaluation

**Figure 6:**
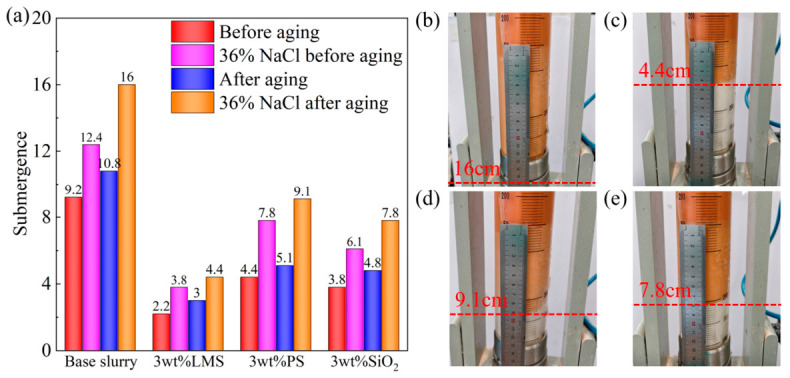
Sand bed plugging test. (**a**) Sand bed invasion depth, (**b**) invasion effect image of base mud (aged at 220 °C/16 h, sat. salt), (**c**) base mud + 3 wt% LMS (aged at 220 °C/16 h, sat. salt), (**d**) base mud + 3 wt% PS (aged at 220 °C/16 h, sat. salt), and (**e**) base mud + 3 wt% SiO_2_ (aged at 220 °C/16 h, sat. salt).

The sand bed plugging performance and invasion depth of the base mud with 3 wt% LMS, PS, and SiO_2_ are shown in Figure 6.

At room temperature, the invasion depth of the base mud was 9.2 cm. Compared to the base mud, the addition of LMS, PS, and SiO_2_ reduced the invasion depth to 2.2 cm, 4.4 cm, and 3.8 cm, respectively. All three gel plugging agents exhibited some plugging effects, with LMS showing the best performance. At a NaCl concentration of 36%, the invasion depth of the base mud was 12.4 cm. Salt ion invasion weakened the effectiveness of the gel plugging agents, but they still retained some plugging ability. Compared to the base mud, the addition of LMS, PS, and SiO_2_ reduced the invasion depth to 3.8 cm, 7.8 cm, and 6.1 cm, respectively. LMS was least affected by salt ions and still exhibited good plugging performance.

At a NaCl concentration of 36%, after aging at 220 °C, the base mud was completely invaded, with an invasion depth of 16 cm. High-temperature and salt ion invasion together weakened the plugging effect. The invasion depths for base mud containing LMS, PS, and SiO_2_ were 4.4 cm, 9.1 cm, and 7.8 cm, respectively. Under conditions of high temperature, 220 °C, and saturated salt, PS and SiO_2_ showed poor plugging performance, while LMS still maintained a good plugging effect, indicating that LMS possesses excellent high-temperature- and high-salinity-resistant plugging performance in drilling fluid base mud.

#### 2.3.3. Core Plugging Evaluation

**Figure 7:**
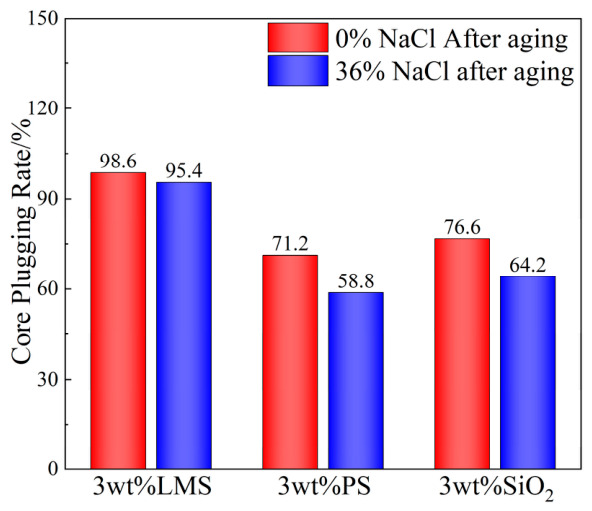
Core plugging test (aged at 220 °C for 16 h).

The results of core plugging tests for drilling fluids with 3 wt% LMS, PS, and SiO_2_ before and after saturated salt addition are shown in Figure 7. (The core is an artificial sandstone core with an average permeability of 10 mD.)

The figure shows that before salt addition, LMS exhibited the best core plugging effect, with a core plugging rate of 98.6%. SiO_2_ showed an intermediate effect (76.6%), and PS showed the poorest effect (71.2%).

After adding saturated salt, the core plugging effect of all three gel plugging agents decreased. However, the core plugging rate for LMS remained high at 95.4%, still demonstrating good core plugging performance. The core plugging rates for SiO_2_ and PS decreased to 64.2% and 58.8%, respectively. This is because PS undergoes enhanced thermal decomposition in high-salinity environments, while silica cannot maintain good dispersion stability, preventing effective entry into and sealing of fractures. In contrast, LMS, with its deformable outer layer and strong internal support, achieves the best plugging effect. This indicates that LMS possesses good core plugging performance under 220 °C high-temperature and saturated-salt conditions, demonstrating excellent temperature and salt resistance.

### 2.4. Mechanism Study

#### 2.4.1. High-Temperature Particle Size Stability Analysis

**Figure 8:**
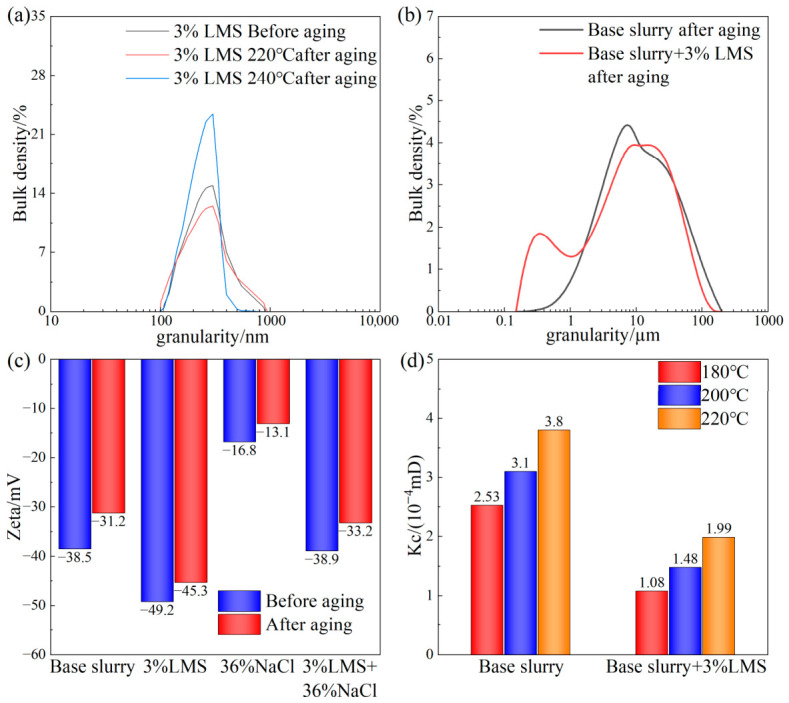
Mechanism analysis of LMS. (**a**) Particle size stability analysis of LMS, (**b**) drilling fluid particle size analysis (aged at 220 °C/16 h, sat. salt), (**c**) zeta potential values, (**d**) mud cake permeability.

Figure 8a shows the particle size distribution of LMS before and after high-temperature aging. After aging at 220 °C, the particle size distribution trend of the microspheres remained essentially unchanged, with only a small number of agglomerated large particles appearing. The particle size of most particles did not change, and the median particle size only increased from 311 nm to 313 nm. When the aging temperature increased to 240 °C, the median particle size only increased to 335 nm, indicating that LMS maintains good particle size stability even at 240 °C.

#### 2.4.2. Analysis of Gel Plugging Agent Effect on Drilling Fluid Particle Size Distribution at High Temperature

Figure 8b shows the particle size distribution change in the saturated brine base mud before and after adding LMS following aging at 220 °C. It can be seen that under high-temperature and high-salinity conditions, LMS supplements the lack of micro-nano particles, optimizing the particle size distribution of the drilling fluid solids to some extent. It fills micro-nano pores in the filter cake, making it denser, which is the direct reason for the reduction in drilling fluid filtration loss.

#### 2.4.3. Zeta Potential Analysis

The zeta potential values of the base mud before and after adding LMS are shown in Figure 8c. When 3 wt% LMS was added to the base mud, the absolute value of the zeta potential of the drilling fluid increased. This is because LMS microspheres carry a large number of negative charges and a small number of positive charges, allowing them to adsorb onto clay surfaces through hydrogen bonding and electrostatic interactions. This brings more hydrated ions to the clay, thereby enhancing the hydration strength and colloidal dispersion stability of the clay. Additionally, the abundant negative charges on LMS itself increase inter-particle repulsion, enhancing particle movement and improving system stability. After adding a large amount of NaCl to the base mud, LMS still effectively enhanced hydration. With the addition of 3 wt% LMS, the zeta potential of the drilling fluid increased to −33.2 mV, indicating that LMS can synergize with clay under high-temperature and high-salinity conditions to resist the destructive effects on the diffuse double layer, effectively improving the colloidal dispersion stability of the drilling fluid.

#### 2.4.4. Mud Cake Permeability Analysis

The permeability of mud cakes after aging at different temperatures for 16 h is shown in Figure 8d. It can be seen that after aging at different temperatures, the mud cake permeability of the sample slurry containing LMS was lower, and the filtrate increase was noticeably slower during testing. This is attributed to the small particles of the LMS gel plugging agent blocking micro-nano pores and fissures in the mud cake, thereby enhancing its compactness and reducing its permeability. After aging at 220 °C for 16 h, the permeability of the mud cake from the drilling fluid base mud was 3.80 × 10^−4^ mD, while the permeability of the mud cake after adding 3% LMS was 1.99 × 10^−4^ mD, indicating that LMS can effectively improve the microstructure of the mud cake and reduce micro-pores and fissures within it.

#### 2.4.5. Mud Cake Micromorphology Analysis

**Figure 9:**
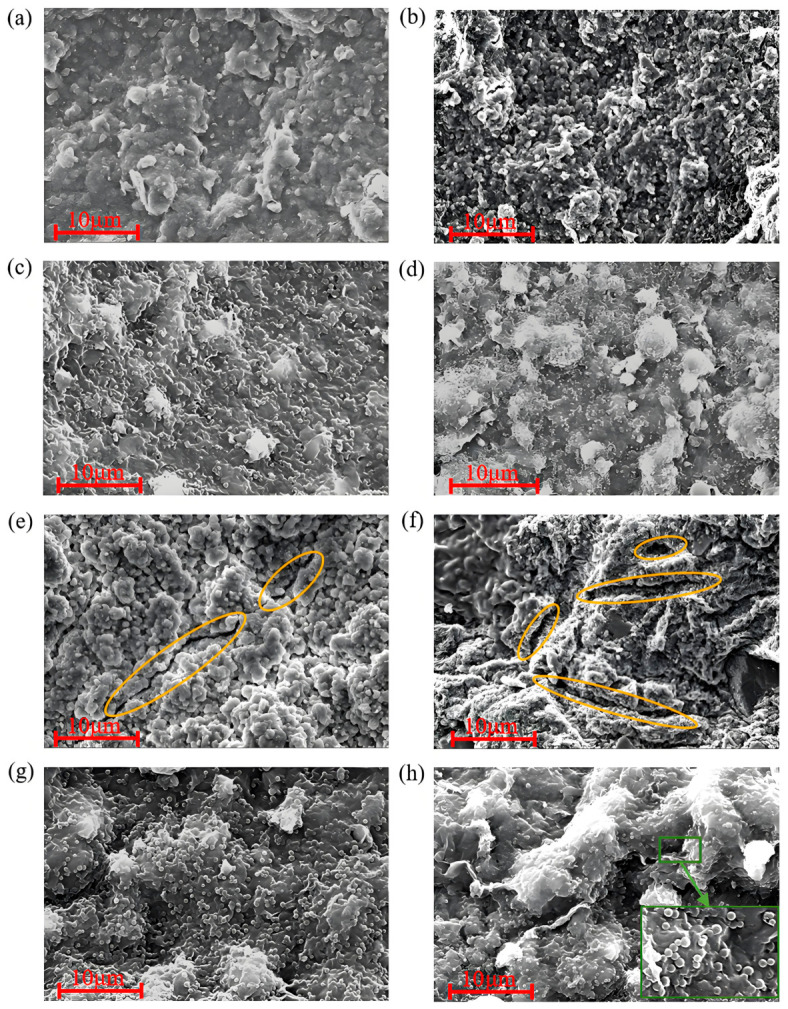
Mud cake morphology analysis. (**a**) Base mud, (**b**) base mud (sat. salt), (**c**) base mud + 3 wt% LMS, (**d**) base mud + 3 wt% LMS (sat. salt), (**e**) base mud (aged 220 °C/16 h), (**f**) base mud (aged 220 °C/16 h, sat. salt), (**g**) base mud + 3 wt% LMS (aged 220 °C/16 h), and (**h**) base mud + 3 wt% LMS (aged 220 °C/16 h, sat. salt).

The surface micromorphology of the mud cakes was observed using SEM, as shown in Figure 9.

Figure 9a,e show the surface morphology of the original drilling fluid base mud cake before and after aging. At room temperature, the mud cake’s surface appears loose and porous, with distinctly distributed platy clay. After aging, the platy structure of the clay is damaged, and cracks are clearly visible on the surface. Figure 9b,f show the surface morphology of the base mud cake with 36% NaCl before and after aging. It can be seen that due to salt ion invasion, clay particles on the mud cake’s surface coalesce into larger aggregates, exhibiting flocculation. After aging, the mud cake’s surface is covered with numerous cracks and pores. Figure 9c,g show the surface morphology of the base mud cake treated with LMS before and after aging. LMS appears as distinct nanospherical particles that can effectively block the pores and channels in the mud cake. The LMS-treated mud cake’s surface is smooth and dense, with significantly fewer channels and gaps. After aging, LMS is still clearly distributed on the mud cake’s surface, effectively sealing surface cracks and making the mud cake relatively flat. Figure 9d,h show the surface morphology of the LMS-treated base mud cake with 36% NaCl before and after aging. It can be seen that after salt ion invasion, clay particles coalesce, but LMS still effectively blocks pores and channels, maintaining a good effect. After aging, LMS is still clearly distributed on the mud cake’s surface, and the mud cake remains flat and dense. The micromorphological analysis of the mud cake demonstrates that LMS can still exert its plugging function effectively under 220 °C high-temperature and saturated-salt conditions.

#### 2.4.6. Mechanism of Action and Potential Analysis

**Figure 10:**
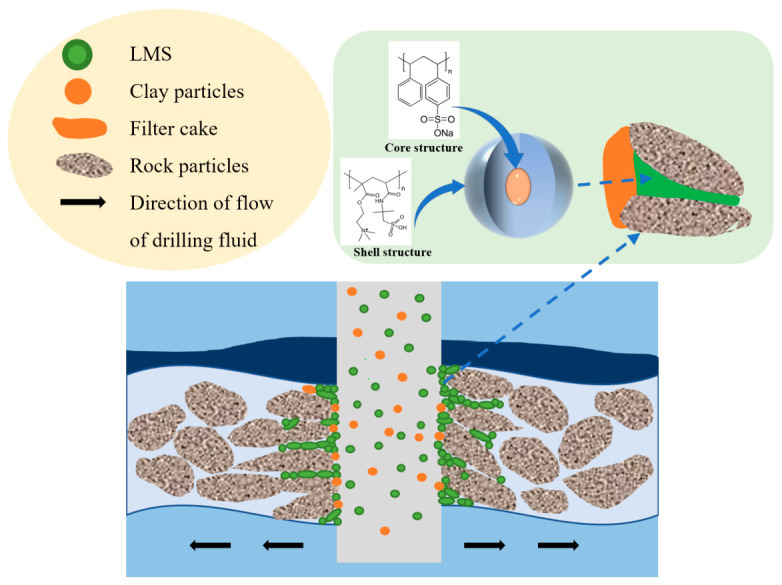
Schematic diagram of LMS plugging.

The mechanism of action of LMS in ultra-high-temperature, high-salinity water-based drilling fluids is shown in Figure 10.

Experiments show that PSDA can form a mud cake together with clay particles. Under differential pressure, PSDA can deform, fill cracks and pores, and continuously accumulate to form a dense plugging layer, preventing further fluid invasion into the cracks and pores. This process indicates that LMS possesses the advantages of both flexible and rigid plugging agents: its internal core structure is rigid, providing good support, while the outer shell is highly flexible, offering strong adaptability to complex formations with micro-pores of varying sizes. LMS exhibits significant potential for application in ultra-high-temperature, complex pore formations.

## 3. Conclusions

1. A gel plugging agent, LMS, with a core–shell structure, resistant to high temperatures up to 240 °C and saturated salt, was prepared using emulsion polymerization. The core is composed of a styrene–sodium p-styrenesulfonate copolymer, and the shell is composed of 2-acrylamido-2-methylpropanesulfonic acid and methacryloyloxyethyltrimethyl ammonium chloride as modifying monomers. The median particle size of LMS is 311 nm. The FTIR spectrum of LMS exhibits characteristic peaks of all monomers. The initial thermal decomposition temperature of LMS is 302.5 °C, indicating good thermal stability. TEM observation revealed a distinct core–shell structure for LMS, confirming successful synthesis and conformity with the expected product.
2. Performance evaluation experiments of LMS were conducted. The results show that LMS possesses good temperature and salt resistance. With 3 wt% LMS and 36 wt% NaCl concentration, after aging at 220 °C for 16 h, the drilling fluid filtration loss was 104.2 mL, a reduction of 57.4% compared to the base mud. LMS can effectively plug microporous membranes with different pore sizes. At a 3 wt% LMS dosage, the sand bed invasion depth of the saturated salt base mud after aging at 220 °C for 16 h was 4.4 cm. At a 3 wt% LMS dosage, the core plugging rate of the saturated salt base mud after aging at 220 °C for 16 h was 95.4%.
3. Compared to the commonly used rigid gel plugging agent SiO_2_ and flexible gel plugging agent PS, LMS exhibits superior filtration reduction and plugging performance under conditions of 220 °C and saturated salt. This indicates that LMS combines the advantages of both rigid and flexible gel plugging agents, i.e., the rigid internal core structure provides good support, while the flexible external shell structure offers strong adaptability to micro-pores of varying sizes, aligning with the design expectations.
4. The mechanism of LMS was revealed the following. High-temperature particle size stability analysis shows that LMS maintains good particle size stability even at an ultra-high temperature of 240 °C. Analysis of the gel plugging agent’s effect on drilling fluid particle size distribution at a high temperature indicates that LMS optimizes the particle size gradation of drilling fluid solids, resulting in a denser filter cake. Zeta potential analysis shows that under 220 °C high-temperature and saturated-salt conditions, LMS can synergize with clay to resist the destruction of its diffuse double layer caused by high temperature and high salinity, effectively improving the colloidal dispersion stability of the drilling fluid. Mud cake permeability analysis shows that under high-temperature conditions of 220 °C, LMS can effectively improve the microstructure of the mud cake, reducing the formation of micro-pores and fissures, and decreasing drilling fluid invasion into the formation. Mud cake micromorphology analysis visually demonstrates that under 220 °C high-temperature and saturated-salt conditions, LMS can still effectively exist on the clay surface and perform its plugging function.

## 4. Materials and Methods

### 4.1. Materials and Instruments

Styrene (St, 98 wt%), sodium p-styrenesulfonate (SSS, 98 wt%), 2-acrylamido-2-methylpropanesulfonic acid (AMPS, 98 wt%), and methacryloyloxyethyltrimethyl ammonium chloride (DMC, 98 wt%) were purchased from Shanghai Macklin Biochemical Technology Co., Ltd. (Shanghai, China). Potassium persulfate (KPS, 98 wt%) and sodium chloride (NaCl, 99.5 wt%) were purchased from Sinopharm Chemical Reagent Co., Ltd. (Shanghai, China). SP-80 (98 wt%) was purchased from Aladdin Biochemical Technology Co., Ltd. (Shanghai, China). Silica (median particle size 300 nm) (SiO_2_, 99 wt%) and polystyrene microspheres (PS, 98 wt%) were purchased from Saan Chemical Technology (Shanghai) Co., Ltd. (Shanghai, China). Drilling fluid bentonite was purchased from Shandong Huawei Bentonite Co., Ltd. (Weifang, Shandong, China).

The main instruments used include: a Fourier transform infrared spectrometer (IRTRacer-100, Shimadzu, Kyoto, Japan), a thermogravimetric analyzer (TGA55, TA Instruments, New Castle, DE, USA), a Transmission Electron Microscope (JEM-ARM300F2, JEOL, Tokyo, Japan), a Six-Speed Rotational Viscometer (ZNN-D6, Qingdao Tongchun, Qingdao, China), an API filter press (SD6A, Qingdao Tongchun, China), Roller Oven (GW300-PLC, Qingdao Tongchun, China), a non-permeation drilling fluid loss tester (FA type, Anton Paar (Shanghai) Trading Co., Ltd., Shanghai, China), and a mud cake thickness and toughness automatic measuring instrument (ZN-1L, Qingdao Tongchun Petroleum Instrument Co., Ltd., Qingdao, China).

### 4.2. Molecular Structure Design of the Gel Plugging Agent

**Figure 11:**
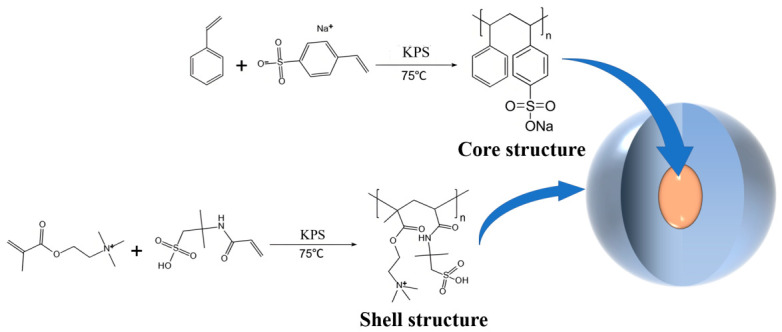
Molecular structure design of the gel plugging agent.

Based on the literature research and analysis of drilling fluid additive failure mechanisms, a design strategy for a high-temperature- and high-salinity-resistant gel plugging agent was devised. Polymer microspheres offer better compatibility in drilling fluids and often possess deformability for better pore plugging. Introducing a high-strength core could further enhance the pressure-bearing capacity of the plugging layer. Accordingly, a polymer microsphere was first prepared as the core, followed by the addition of modifying monomers as the shell, forming a core–shell structured nanosphere with improved compatibility and pore-plugging ability. Following this concept, a nanosphere molecular structure was designed (As shown in Figure 11), consisting of a styrene–sodium p-styrenesulfonate copolymer core coated with a shell of 2-acrylamido-2-methylpropanesulfonic acid and a methacryloyloxyethyltrimethyl ammonium chloride copolymer.

The core formed by copolymerizing sodium p-styrenesulfonate and styrene provides good mechanical strength [23], and the rigidity of the benzene rings can enhance the thermal decomposition temperature of the microsphere, effectively supporting fractures under high-temperature conditions. The flexible shell, composed of AMPS and DMC copolymers, exhibits good flexibility, offering strong adaptability to micro-pores of various sizes. The introduction of cationic groups can enhance the adsorption capacity of the gel plugging agent onto negatively charged minerals in the formation, synergistically improving the robustness of the plugging layer. In summary, the core–shell structure of LMS enables it to combine the advantages of both rigid and flexible materials.

### 4.3. Synthesis of the Gel Plugging Agent

1. First, 0.5 g of emulsifier SP-80, 30 g of styrene and 15 g of sodium p-styrenesulfonate were dispersed in 100 mL of deionized water, and the mixture was sheared and emulsified using a shear emulsifier at 2000 r/min for 20 min. The dispersion was transferred to a three-necked flask (stirring rate of 450 r/min and reaction temperature of 75 °C) and initiator solution 1 (2 mL water + 0.185 g potassium persulfate) was added. The reaction was carried out under constant temperature for 10 h and then naturally cooled to room temperature to obtain the core-structure plugging agent dispersion.
2. Then, 5 g of 2-acrylamido-2-methylpropane sulfonic acid (AMPS) and 3 g of methacrylatoethyl trimethyl ammonium chloride were dispersed in 15 mL of deionized water, and initiator solution 2 (2 mL water + 0.045 g potassium persulfate) was added and fully stirred to obtain the modifying monomer solution.
3. The core-structure dispersion was heated to 70 °C, then purged with nitrogen and stirred under constant temperature for 20 min; the modifying monomer solution was then slowly added dropwise to the three-necked flask, and the reaction was continued under a constant temperature for 6 h to obtain the final core–shell structural plugging agent.

### 4.4. Characterization

1. Fourier Transform Infrared Spectroscopy (FTIR) [36]: For infrared spectroscopy analysis, a semi-transparent thin tablet of LMS prepared by the KBr pellet method was placed in a Fourier transform infrared spectrometer, and the Fourier transform infrared absorption (FTIR) spectrum of LMS was measured in the range of 4000 to 1000 cm^−1^.
2. Thermogravimetric Analysis (TGA) [37]: A crucible containing LMS was placed in a thermogravimetric analyzer, and the thermogravimetric curve of LMS was measured under a nitrogen atmosphere in the range of 40 to 800 °C at a heating rate of 10 °C/min;
3. Transmission Electron Microscopy (TEM) analysis [38]: LMS microspheres dispersed in deionized water were collected with a copper grid and naturally air-dried, and the microstructure of LMS was observed by transmission electron microscopy.
4. Particle Size Analysis: The particle size of a 3 wt% LMS dispersion in deionized water was measured using an ultra-high-speed laser particle size analyzer, and the particle size distribution curve of LMS at room temperature was plotted.

### 4.5. Evaluation of Rheological and Filtration Properties

Preparation of Base Mud: Prepare a base mud with 4% bentonite content [39].

Investigation of Basic Properties, Temperature Resistance, and Salt Resistance of LMS: Investigate the apparent viscosity (AV), plastic viscosity (PV), yield point (YP), and API filtration loss (FL_API_) of LMS before and after aging at different dosages, different aging temperatures, and different salt concentrations.

Commonly used rigid gel plugging agent silica (SiO_2_) and flexible gel plugging agent polystyrene (PS) were also subjected to the above tests simultaneously for rheological and filtration performance comparison with LMS.

### 4.6. Evaluation of Plugging Performance

1. Microporous Membrane Plugging Performance Evaluation: Four types of polytetrafluoroethylene (PTFE) microporous membranes with different pore sizes were selected. These PTFE microporous membranes are hydrophilic and pressure-resistant, with pore sizes of 100, 300, 500, and 1000 nm, defined as PTFE-100, PTFE-300, PTFE-500, and PTFE-1000 for convenience. The plugging performance of 3 wt% gel plugging agent aqueous solutions against microporous membranes after aging at different temperatures was evaluated. The standard API filter paper for drilling fluids was replaced with the PTFE microporous membrane, and the measurement was conducted following the method for room-temperature API filtration loss (25 °C and 100 psi).
2. Sand Bed Plugging Performance Evaluation: Sand bed plugging performance was tested using a non-permeation drilling fluid loss tester (FA type). The bottom of the transparent cylinder is a filter screen without filter paper. First, 350 mL of 80–120 mesh quartz sand was poured into the transparent cylinder in three portions, manually vibrated and compacted to level the sand surface. Then, 150 mL of drilling fluid with 3 wt% gel plugging agent was poured into the cylinder. The top lid was sealed, and the gas path pressure was adjusted to 0.69 MPa. The gas source was turned on, and timing started. The invasion depth of the drilling fluid was measured after 30 min.
3. Core Plugging Performance Evaluation: Take 400 mL of drilling fluid with 3 wt% gel plugging agent, add NaCl according to test requirements, stir at 5000 rpm for 20 min, place into an aging cell, and put into a roller oven. After rolling at 220 °C for 16 h, remove and cool to room temperature. Stir again at 5000 rpm for 20 min. Test the core plugging rate as follows: Measure the initial positive directional standard brine permeability *K*_1_ of the rock sample using a drilling fluid contamination holder on a core flow experimental apparatus. Then, remove the core contamination holder and connect it to a high-temperature high-pressure dynamic drilling fluid testing instrument. Use the prepared drilling fluid to positively plug the rock sample under the conditions of a drilling fluid temperature of 80 °C, differential pressure of 3.5 MPa, confining pressure of 5 MPa, and shear rate of 150 s^−1^ for a damage time of 30 min. Remove the core contamination holder, reconnect it to the core flow experimental apparatus, and measure the positive directional standard brine permeability *K*_2_ of the rock sample. The core plugging rate *R* is calculated using Equation (1):

(1) $$R=\left(1-\frac{K_{2}}{K_{1}}\right)\times 100\%$$

SiO_2_ and PS were also subjected to the above tests simultaneously for plugging performance comparison with LMS.

### 4.7. Mechanism Study

1. High-Temperature Particle Size Stability Analysis: A 3 wt% LMS dispersion in deionized water was aged at 220 °C and 240 °C, and the particle size distribution of the dispersion was measured using a laser particle size analyzer.
2. Analysis of Gel Plugging Agent Effect on Drilling Fluid Particle Size Distribution at High Temperature: Particle size tests were conducted on drilling fluids with different gel plugging agent dosages using an ultra-high-speed laser particle size analyzer, and particle size distribution curves were plotted.
3. Zeta Potential Analysis: The zeta potential of the drilling fluid diluted at a volume ratio of 1:1000 was measured using a zeta potential analyzer.
4. Mud Cake Permeability Analysis: The permeability of the mud cake was calculated using the Darcy equation [40] (Equation (2)) based on API filter press data. The API filtration differential pressure is 0.69 MPa, the liquid used is deionized water, the filtration rate is the average value calculated from the 30 min filtrate volume, and the mud cake thickness was measured using a mud cake thickness and toughness automatic measuring instrument (ZN-1L, Qingdao Tongchun Petroleum Instrument Co., Ltd.).

(2) $$K_{c}=\frac{\mu t_{c}q}{\Delta PA}$$

where *K_c_*—permeability, mD;

*µ*—filtrate viscosity, mPa·s;

*t_c_*—mud cake thickness, cm;

*q*—filtration rate, cm^3^·s^−1^;

Δ*P*—differential pressure, 10^5^ Pa;

*A*—filtration area, cm^2^.

The permeability of API filter cakes after aging at 180 °C, 200 °C, and 220 °C for 16 h was determined. The viscosity of deionized water was taken as 1 mPa·s, the differential pressure was 6.9 × 10^5^ Pa, and the filtration area was 45.8 cm^2^.

Mud Cake Micromorphology Analysis: API filter cakes were collected from the unaged base mud and drilling fluids containing 3 wt% LMS, as well as from drilling fluids after aging at 220 °C/16 h. The mud cakes were dried, and the surface micromorphology of the mud cakes was observed using a scanning electron microscope (SEM).

## Data Availability

The data presented in this study are available on request from the corresponding author.
